# Prevalence and correlates of HPV among women attending family-planning clinics in Thailand

**DOI:** 10.1186/s12879-015-0886-z

**Published:** 2015-03-27

**Authors:** Morgan A Marks, Swati Gupta, Kai-Li Liaw, Amha Tadesse, Esther Kim, Chailert Phongnarisorn, Virach Wootipoom, Pissamai Yuenyao, Charoen Vipupinyo, Sungwal Rugpao, Somchai Sriplienchan, Patti E Gravitt, David D Celentano

**Affiliations:** Merck Research Laboratories, West Point, PA USA; PRA Health Sciences, Fort Washington, PA USA; Johns Hopkins Bloomberg School of Public Health, Baltimore, MD USA; Chiang Mai University, Chiang Mai, Thailand; Prince of Songkla University, Songkla, Thailand; Khon Kaen University, Khon Kaen, Thailand; Rajavithi Hospital, Bangkok, Thailand; Research Institute for Health Sciences, Chiang Mai, Thailand

**Keywords:** HPV, Epidemiology, Thailand, CIN

## Abstract

**Background:**

Cervical cancer is the most common cancer among women of reproductive age in Thailand. However, information on the prevalence and correlates of anogenital HPV infection in Thailand is sparse.

**Methods:**

HPV genotype information, reproductive factors, sexual behavior, other STI and clinical information, and cervical cytology and histology were assessed at enrollment among one thousand two hundred and fifty-six (n = 1,256) HIV negative women aged 20–37 from Thailand enrolled in a prospective study of the natural history of HPV. The type-specific prevalence of HPV was estimated using cervical swab specimens from healthy women and women with a diagnosis of CIN 2/3 at baseline. Prevalence ratios (95% CI) were estimated using Poisson regression to quantify the association of demographic, behavioral, and clinical correlates with prevalent HPV infection.

**Results:**

Overall, 307 (24.6%) and 175 (14.0%) of women were positive for any HPV type and any HR-HPV type, respectively; the most common types were 72, 52, 62, and 16. Among women diagnosed with CIN 2/3 at enrollment (n = 11), the most prevalent HPV types were 52 and 16. In multivariate analysis, HPV prevalence at enrollment was higher among women with: long-term combined oral contraceptive use, a higher number of lifetime sexual partners, a prior Chlamydia infection, and a current diagnosis of Bacterial Vaginosis.

**Conclusion:**

The study findings provide important information that can be used in the evaluation of primary and secondary interventions designed to reduce the burden of cervical cancer in Thailand.

**Electronic supplementary material:**

The online version of this article (doi:10.1186/s12879-015-0886-z) contains supplementary material, which is available to authorized users.

## Background

Cervical cancer affects nearly half a million women worldwide, making it the third most common cancer. Between the years 1983 and 2002, Thailand had a reported average annual cervical cancer incidence rate of 65.2 cases per 100,000 over the 20 year period making it the most common cancer among women between 30–74 years of age with a 2.6% per year increase in incidence [1]. Infection with anogenital types of the human papillomavirus (HPV) is the established cause of cervical cancer [2,3]. HPV types 16 and 18 account for nearly 70% of the total cervical cancer cases detected worldwide with HPV 16 alone accounting for approximately 50% [4].

In a prior population-based study in two districts in northern and southern Thailand, HPV types other than type 16 were as or more prevalent in women both with and without cervical disease [5]. However, this prior work provided only a limited view of the total HPV burden as it included women from only 2 regions in Thailand. Given the large burden of cervical cancer in Thailand, it is important to further understand the HPV type-specific distribution in cervical infections in other regions and populations. This information may help direct appropriate strategies for current and future interventions.

The current study assessed the prevalence and risk factors for anogenital HPV infection and CIN 2/3 in women aged 20–37 years recruited from family-planning clinics in 7 sites throughout the country.

## Methods

### Study population and enrollment

Women 20–37 years old attending family planning clinics in the northern (Chiang Mai), northeastern (Khon Kaen), central (Bangkok) and southern (Songkla/HatYai) regions in Thailand between 2002–2003 were recruited into a prospective study investigating the natural history of HPV and CIN 2/3. These women were previously enrolled in a two-year study addressing the effects of hormonal contraceptive use on HIV acquisition (HC-HIV). Selection criteria are described in detail elsewhere [6]. Briefly, inclusion criteria for enrollment in the HC-HIV study were: 1) HIV negative; 2) not pregnant; 3) intact uterus; 4) used some form of contraception in the 3 months prior to enrollment; and 5) willing to adhere to the self-selected contraceptive method for at least 1 year of follow-up. Among women who participated in the HC-HIV study, 79% were reconsented for inclusion in the current study (n = 1256). The study protocols were reviewed and approved by the committees on human subject research at Johns Hopkins Bloomberg School of Public Health, Baltimore, MD, Merck Research Laboratories, West Point, PA, each study recruitment site, and the Institutional Review Board of the Thailand Ministry of Health (MOH), Thailand.

At enrollment, information on sociodemographic characteristics, sexual risk behavior, reproductive and contraceptive history, current contraceptive usage status, self-reported medical history, and woman’s report of the sexual behavior of her partner was collected at each study site by trained interviewers using a standardized questionnaire.

Sexual behavior variables included age of sexual debut, lifetime number of sexual partners, number of sexual partners in the last six months (L6M) and/or new sexual partners acquired in the last year (L12M), commercial sex work L6M, condom use with primary partner L6M or most recent sexual partner if no primary partners are reported by the participant, and primary partner risk behaviors. Reproductive information included total number of pregnancy and live births. Contraceptive use was broadly classified as 1) combined low-dose oral contraceptives (COC); 2) depomedroxyprogesterone acetate (DMPA); 3) other injectable methods; and 4) non-hormonal contraceptive use and non-use (NHC). Current use and duration of use prior to enrollment for each category of hormonal contraception were assessed.

Questionnaire and physical exam data from the original HC-HIV study were extracted and linked to the participants. Laboratory-confirmed STI status was collected from the two year period prior to study enrollment and included gonorrhea (GC), chlamydia (CT), syphilis (SYP), and Bacterial Vaginosis (BV).

### Physical examination and specimen collection

At enrollment, each participant underwent a pelvic examination. Exfoliated cervical cells were collected using a cytobrush and placed in PreservCyt™ for Thinprep™ liquid-based cytology. CT and GC detection from an ectocervical mucus specimen was performed using the Roche Amplicor assay per manufacturer’s instructions (Roche Molecular Systems, Alameda, CA). An endo/ecto cervical swab specimen was collected by study clinicians for HPV DNA genotyping using a Dacron swab stored in Specimen Transport Medium (STM) (Digene) at −20^○^C until testing. Bacterial Vaginosis was diagnosed by the Ames test.

All Pap smear and biopsy specimens were read by trained cytopathologists (Covance, Indianapolis, IN). Cytological smears were classified according to the Bethesda system [7]. Participants with an abnormal Pap smear diagnosis of atypical squamous cell of undetermined significance (ASC-US) or more severe (≥ ASC-US) were referred for colposcopic examination with biopsy and treatment as indicated. Colposcopy directed biopsies were reported as a diagnosis of Normal, Cervical Intraepithelial Neoplasia 1 (CIN 1), Cervical Intraepithelial Neoplasia II (CIN 2), and Cervical Intraepithelial Neoplasia 3 (CIN 3). Biopsies were read and interpreted by one study pathologist.

### HPV DNA testing

All HPV DNA testing was performed on cervical cell samples stored in STM (Digene) at Johns Hopkins University, Baltimore, MD. DNA was extracted using the QIAamp DNA Blood Kit (Qiagen, Courtaboeuf, France) according to manufacturer’s instructions with modification. After extraction, DNA was tested using the Roche HPV Linear array© PCR assay (Roche Diagnostics, Indianapolis, IN). The HPV Linear Array^©^ is based on the PGMY09/11 primer system that allows for high efficiency amplification of 37 types of HPV [8,9]. The quality and validity of the extracted DNA specimen was assessed by inclusion of β-globin gene-specific primers in the PCR reaction; only specimens with detectable β-globin were used in this analysis.

HPV types considered to be high risk (HR-HPV) for this analysis included 16, 18, 31, 33, 35, 39, 45, 51, 52, 56, 58, 59, 68, 73, 82 [3,10]. Low risk (LR) HPV types included all other HPV types detected by the HPV Linear Array^©^. Multiple infections that include a HR-HPV type were classified as high risk regardless of the presence of other low risk co-infections.

### Statistical analysis

The prevalence of HPV any, high-risk HPV (HR-HPV), low-risk HPV (LR-HPV), and type-specific infection was computed among the total study sample and among those with a diagnosis of CIN 2/3 at enrollment.

Contingency tables were created to assess the distribution of demographic, sexual, clinical, and reproductive factors by detection of HPV DNA. Due to the high prevalence of HPV in the study sample, univariate prevalence ratios (PR) were estimated using a generalized linear model with a Poisson function and robust variance to assess the strength of the association between a given risk factor and detection of any HPV or any HR-HPV type [11].

Covariates with a p-value of <0.1 in univariate analyses were considered for inclusion in the multivariate regression model. Following this initial selection, variables were removed from the model in a stepwise fashion and a likelihood ratio test was performed after removal of each variable to confirm the variables contribution to the model’s goodness-of-fit and to identify the most parsimonious model. Age and study site were retained in the multivariate model. After identifying the final multivariate model using an outcome of any HPV infection, separate multivariate models were then generated that assessed the relationship of the identified demographic, behavioral and clinical factors on other outcomes such as (1) any HR-HPV infections; (2) HPV 52 infections; and (3) HPV 52 infections without HPV16 infections. The statistical significance of trends for PRs was assessed by including a categorical variable as a continuous variable in the regression model. The final model for any HPV infection was stratified by cytological diagnosis at baseline (Normal vs. Abnormal [including inflammation, ASCUS, LSIL, and HSIL]). A p-value of <0.05 was considered statistically significant in the final multivariate model for the association of a given variable and detection of HPV DNA. All analyses were conducted using STATA 11.0 (STATACORP, College Station, TX).

## Results

At enrollment, 6 (0.4%) of the 1256 samples were β-globin negative leaving a total of 1250 samples for analysis. Overall, 307 (24.6%) and 175 (14.0%) of women were positive for any HPV or any HR-HPV type (57% of HPV DNA positive women), respectively (Table 1). There were 94 (7.6%) of women with multiple HPV infections (30.9% of HPV-positive women). The three most common types found in either a single or multiple infection were the low risk types 72 (4.1%) and 62 (2.9%) as well as the high-risk type 52 (3.6%). A total of 11 women had a histological diagnosis of CIN2/3 at enrollment. Among women diagnosed with CIN 2/3, the prevalence of any HPV type and any HR-HPV type was 90.9% for both categories. The two most prevalent HPV types detected among women with CIN 2/3 was HPV 52 (63.6%) and HPV 16 (27.3%).

**Table 1:** **Prevalence of cervical HPV infections overall and among those with prevalent CIN 2/3 cases**

**Type**	**Prevalence (N = 1250)**	**Prevalence among those with prevalent CIN 2/3 (n = 11)**	
	**n(%)**	**n (%)**	**(95%** **CI)**
HPV DNA negative	943	1 (9.1)	(0.2, 41.3)
Any HPV positive	307 (24.6)	10 (90.9)	(58.7, 99.8)
Any HR-HPV positive	175 (14)	10 (90.9)	(58.7, 99.8)
Any LR-HPV positive	170 (13.6)	2 (18.2)	(2.3, 51.8)
High-Risk Infections:			
HPV 16	26 (2.1)	3 (27.3)	
HPV 18	9 (0.7)	2 (18.2)	(2.3, 51.8)
HPV 31	7 (0.6)	0	-----
HPV 33	7 (0.6)	1 (9.1)	(0.7, 20.2)
HPV 35	3 (0.2)	0	-----
HPV 39	20 (1.6)	1 (9.1)	(0.2, 41.3)
HPV 45	3 (0.2)	1 (9.1)	(0.2, 41.3)
HPV 51	23 (1.8)	0	-----
HPV 52	45 (3.6)	7 (63.6)	(30.8, 89.1)
HPV 56	6 (0.5)	0	-----
HPV 58	11 (0.9)	0	-----
HPV 59	12 (0.9)	0	-----
HPV 68	21 (1.7)	1 (9.1)	(0.2, 41.3)
HPV 73	6 (0.5)	0	-----
HPV 82	6 (0.5)	0	-----
Low-Risk Infections:			
HPV 6	1 (0.1)	0	-----
HPV 11	1 (0.1)	0	-----
HPV 26	0	0	-----
HPV is39	2 (0.2)	0	-----
HPV 40	4 (0.3)	0	-----
HPV 42	6 (0.5)	0	-----
HPV 53	27 (2.2)	0	-----
HPV 54	13 (1.0)	0	-----
HPV 55	8 (0.6)	0	-----
HPV 61	3 (0.2)	0	-----
HPV 62	36 (2.9)	0	-----
HPV 64	3 (0.2)	0	-----
HPV 66	7 (0.6)	0	-----
HPV 67	2 (0.2)	1 (9.1)	(0.2, 41.3)
HPV 69	1 (0.1)	0	-----
HPV 70	27 (2.2)	0	-----
HPV 71	22 (1.8)	0	-----
HPV 72	51 (4.1)	1 (9.1)	(0.2, 41.3)
HPV 81	9 (0.7)	0	-----
HPV 83	1 (0.1)	1 (9.1)	(0.2, 41.3)
HPV 84	14 (1.1)	0	-----
HPV 89	4 (0.3)	0	-----
HPV 16 (w/o HPV 52)	22 (1.8)	1 (9.1)	(0.2, 41.3)
HPV 52 (w/o HPV 16)	41 (3.5)	5 (45.5)	(16.7, 76.6)
Other HR-HPV types (w/o HPV 16/52)	105 (10.6)	2 (18.2)	(2.3, 51.8)
LR-HPV (w/o HR-HPV)	115 (8.4)	2 (18.2)	(2.3, 51.8)
Number of unique HPV types detected:			
1	212 (16.9)	4 (36.4)	(10.9, 69.2)
2	62 (4.9)	3 (27.3)	(6.0, 60.9)
3	22 (1.8)	3 (27.3)	(6.0, 60.9)
4	7 (0.6)	0	-----
5	4 (0.3)	0	-----

### Correlates of prevalent HPV infection

At enrollment, 33 (2.6%) women had missing parity data, 1 (0.1%) woman had a missing STI diagnosis data, and 5 women who reported use of other injectable contraceptives were excluded from the analysis leaving 1,201 women to examine the association of risk factors for prevalent HPV infection.

The prevalence of an HPV infection was significantly lower among women reporting one or more live births as compared to those with none (24.5% vs. 22.1%; p < 0.001) (Table 2). The prevalence of any HPV was significantly higher among women reporting greater than 6 years of COC use as compared to never users (31.5% vs. 21.1%; p < 0.001). The prevalence of any HPV was significantly higher among current smokers as compared to non-smokers (56.4% vs. 22.5%; p < 0.001). Lastly, a higher HPV prevalence was observed among women with risker sexual behavior such as younger age of sexual debut, increased number of recent and lifetime partners, and women reporting commercial sex work. Similar associations with demographic and reproductive factors were observed for HR-HPV infections.

**Table 2:** **Univariate association of demographic information and reproductive history with prevalent infection of any HPV and any HR-HPV type**

**Variable**	**Sample N = 1,201**	**HPV positive n = 289**	**Unadjusted prevalence ratio**	**HR-HPV positive N = 163**	**Unadjusted prevalence ratio**
		**(24.1%** **)**	**(95%** **CI)**	**(13.6%** **)**	**(95%** **CI)**
Age category, years					
<26	224	26.3%	1.0	18.3%	1.0
26-30	422	22.8%	0.86 (0.65, 1.14)	12.6%	0.69 (0.47, 0.99)
31-33	279	25.1%	0.95 (0.71, 1.28)	11.8%	0.65 (0.42, 0.99)
34-38	276	23.2%	0.89 (0.65, 1.19)	13.0%	0.71 (0.47, 1.08)
Study Site in Thailand					
North	429	20.5%	1.0	10.5%	1.0
North-East	274	21.2%	1.03 (0.77, 1.38)	11.7%	1.11 (0.73, 1.71)
South	311	27.9%	1.36 (1.05, 1.77)	16.4%	1.56 (1.08, 2.27)
Central	187	29.9%	1.46 (1.09, 1.95)	18.7%	1.78 (1.19, 2.68)
Years of education					
>12	149	28.9%	1.0	14.8%	1.0
10-12	235	20.4%	0.71 (0.49, 1.01)	10.2%	0.69 (0.40, 1.19)
7-9	277	25.9%	0.90 (0.65, 1.24)	15.5%	1.05 (0.65, 1.69)
≤6	540	23.3%	0.81 (0.60, 1.09)	13.7%	0.93 (0.59, 1.44)
# of pregnancies:					
0-1	444	23.2%	1.0	14.4%	1.0
2	500	23.2%	1.00 (0.79, 1.26)	12.4%	0.86 (0.62, 1.19)
≥3	257	27.2%	1.17 (0.90, 1.53)	14.4%	0.99 (0.69, 1.45)
# of livebirths					
0	27	55.6%	1.0	22.2%	1.0
1	604	24.5%	0.44 (0.31, 0.64)	15.7%	0.71 (0.34, 1.47)
>1	570	22.1%	0.39 (0.27, 0.58)	10.9%	0.49 (0.23, 1.03)
Contraceptive use at enrollment*:					
NHC	448	24.8%	1.0	12.5%	1.0
DMPA	347	19.9%	0.80 (0.62, 1.05)	11.8%	0.95 (0.65, 1.38)
COC	406	26.9%	1.08 (0.86, 1.36)	16.3%	1.30 (0.94, 1.81)
Cumulative use of COCs:					
Never	194	21.1%	1.0	12.4%	1.0
<4 years	603	22.6%	1.07 (0.78, 1.45)	11.8%	0.95 (0.62, 1.47)
4-6 years	310	23.6%	1.11 (0.79, 1.56)	13.6%	1.09 (0.69, 1.75)
>6 years	94	31.5%	1.96 (1.37, 2.82)	27.7%	2.24 (1.36, 3.68)
Cumulative use of DMPA:					
Never	272	27.6%	1.0	15.1%	1.0
<4 years	558	25.3%	0.92 (0.72, 1.16)	13.9%	0.93 (0.65, 1.32)
4-6 years	315	18.7%	0.68 (0.50, 0.92)	11.8%	0.78 (0.52, 1.18)
>6 years	56	25.0%	0.91 (0.55, 1.48)	12.5%	0.83 (0.39, 1.75)
Current Smoker:					
No	1146	22.5%	1.0	12.5%	1.0
Yes	55	56.4%	2.50 (1.94, 3.23)	36.4%	2.91 (1.99, 4.27)
Age of sexual debut, years:					
>20	459	20.3%	1.0	10.2%	1.0
17-19	550	23.6%	1.17 (0.92, 1.48)	13.6%	1.33 (0.94, 1.88)
<17	192	34.4%	1.69 (1.29, 2.22)	21.4%	2.09 (1.42, 3.06)
Lifetime # of sex partners:					
1	852	17.4%	1.0	9.7%	1.0
2	179	31.8%	1.83 (1.41, 2.38)	13.9%	1.43 (0.94, 2.18)
3	61	32.8%	1.89 (1.28, 2.78)	18.0%	1.85 (1.04, 3.28)
≥4	109	58.7%	3.38 (2.73, 4.19)	40.4%	4.14 (3.05, 5.63)
# Partners L6M**:					
0	29	10.3%	1.0	3.5%	1.0
1	1,133	22.8%	2.20 (0.75, 6.46)	12.4%	3.58 (0.52, 24.8)
>1	39	71.8%	6.94 (2.33, 20.6)	56.4%	16.4 (2.34, 114.6)
New partner L12M:***					
No	1128	22.7%	1.0	12.5%	1.0
Yes	44	68.2%	3.00 (2.39, 3.78)	47.7%	3.82 (2.70, 5.39)
Commercial Sex Work L6M :					
No	1167	22.7%	1.0	12.3%	1.0
Yes	34	70.6%	3.11 (2.44, 3.96)	55.9%	4.53 (3.24, 6.34)
Condom use L6M:					
No	977	20.9%	1.0	11.6%	1.0
Yes	224	37.5%	1.79 (1.45, 2.20)	22.3%	1.93 (1.43, 2.60)
Primary partner L6M:					
Yes	1164	23.9%	1.0	13.5%	1.0
No	8	87.5%	3.65 (2.76, 4.84)	62.5%	4.63 (2.66, 8.08)
Primary partner had sex with other^†^ L6M:****					
No	902	20.6%	1.0	11.2%	1.0
Yes	76	48.7%	2.36 (1.81, 3.07)	30.3%	2.70 (1.83, 3.98)
Don’t Know	186	30.1%	1.46 (1.13, 1.88)	17.7%	1.58 (1.11, 2.27)

*NHC = Non-Hormonal Contraception; DMPA = DepotMedroxyprogesterone Acetate; COC = Combined oral contraception **L6M = Last six months prior to enrollment; L12M = Last twelve months prior to enrollment ***Among those who report > =1 sexual partner L6M ****Among those who report a primary partner L6M ^†^Includes commercial and non-commercial sexual partners.

A higher prevalence of any HPV was observed in women with a cytological diagnosis of inflammation (43.8%), AS-CUS (36.5%), LSIL (80.9%), or HSIL (71.4%) as compared to normal (20.3%) (p < 0.001) (Table 3). A higher prevalence of HPV was observed among women with a prior diagnosis of genital ulcers (36.2% vs. 22.0%; p < 0.001) and genital warts (42.2% vs. 23.5%; p = 0.01). No women had genital warts or ulcers upon physical examination at study enrollment. Women with a prior and current diagnosis of gonnorhea, chlamyida, and bacterial vaginosis had a significantly higher prevalence of any HPV infection. Similar associations were observed with HR-HPV infections.

**Table 3:** **Univariate association of cytological diagnosis, clinical and STI history with prevalent infection with any HPV and any HR-HPV type**

**Variable**	**Sample N = 1201**	**HPV + ve N = 289**		**HR-HPV + ve N = 163**	
		**(24.1%)**	**uPR (95%** **CI)**	**(13.6%** **)**	**uPR (95%** **CI)**
Pap smear diagnosis:					
Normal	1077	20.3%	1.0	10.3%	1.0
Inflammation	16	43.8%	2.15 (1.22, 3.79)	25.0%	2.43 (1.02, 5.77)
AS-CUS	52	36.5%	1.79 (1.23, 2.62)	28.9%	2.79 (1.76, 4.44)
LSIL	42	80.9%	3.98 (3.30, 4.81)	57.1%	5.54 (4.04, 7.60)
HSIL	14	71.4%	3.51 (2.47, 4.99)	64.3%	6.24 (4.06, 9.57)
Genital ulcer ever:					
No	1027	22.0%	1.0	12.5%	1.0
Yes	174	36.2%	1.64 (1.31, 2.07)	20.1%	1.61 (1.15, 2.26)
Genital warts ever:					
No	1168	23.5%	1.0	13.4%	1.0
Yes	33	42.2%	1.80 (1.19, 2.72)	18.2%	1.35 (0.65, 2.83)
PID ever:					
No	1151	23.6%	1.0	13.5%	1.0
Yes	50	34.0%	1.44 (0.96, 2.15)	16.0%	1.19 (0.62, 2.28)
Ever Gonnorhea:					
No	1162	23.2%	1.0	12.6%	1.0
Yes	39	48.7%	2.10 (1.49, 2.94)	43.6%	3.47 (2.35, 5.11)
Ever Chlamydia:					
No	1044	21.7%	1.0	11.3%	1.0
Yes	157	40.1%	1.85 (1.48, 2.32)	28.7%	2.54 (1.88, 3.42)
Ever Syphilis:					
No	1183	24.0%	1.0	13.5%	1.0
Yes	18	27.8%	1.16 (0.55, 2.45)	16.7%	1.23 (0.43, 3.49)
Current gonnorhea:					
No	1199	24.0%	1.0	13.5%	1.0
Yes	2	50.0%	2.08 (0.52, 8.36)	50 .0%	3.70 (0.92, 14.9)
Current Chlamydia:					
No	1186	23.6%	1.0	13.1%	1.0
Yes	15	60.0%	2.54 (1.66, 3.89)	53.3%	4.08 (2.49, 6.7)
Ever Bacterial Vaginosis.:					
No	980	21.5%	1.0	11.9%	1.0
Yes	221	35.3%	1.64 (1.32, 2.03)	20.8%	1.74 (1.28, 2.37)
Current Bacterial Vaginosos.:					
No	1142	22.8%	1.0	12.7%	1.0
Yes	59	49.2%	2.16 (1.63, 2.86)	30.5%	2.40 (1.59, 3.64)

In multivariate analysis (Table 4), prevalent infection with any HPV and any HR-HPV was associated with primary partner sexual behavior, >6 years cumulative use of COCs, an increased number of lifetime partners, prior detection of Chlamydia, and current diagnosis of bacterial vaginosis. The magnitude of the prevalence ratios were similar when analyses were restricted to individuals with HPV 52 infections with or without concurrent HPV 16 infections but the associations did not reach statistical significance due to reduced sample size. There was little to no difference in prevalence ratios among women with and without cytological abnormalities (Additional file 1: Table S1).

**Table 4:** **Multivariate association of factors with prevalent infection of any HPV and any HR-HPV**

**Variable**	**Adjusted PR (95%** CI)**			
	**Any HPV**	**Any HR-HPV**	**HPV 52**	**HPV 52 (w/o HPV 16)**
Age at enrollment:				
<26	1.0	1.0	1.0	1.0
26-30	0.89 (0.68, 1.16)	0.73 (0.49, 1.06)	0.99 (0.46, 2.11)	0.99 (0.46, 2.11)
31-33	1.02 (0.77, 1.37)	0.72 (0.47, 1.09)	0.51 (0.19, 1.35)	0.51 (0.19, 1.35)
34-38	0.95 (0.70, 1.29)	0.73 (0.48, 1.13)	0.35 (0.12, 1.03)	0.35 (0.12, 1.03)
Cumulative use of COCs*:				
Never	1.0	1.0	1.0	1.0
<4 years	1.17 (0.87, 1.58)	1.06 (0.71, 1.59)	0.50 (0.22, 1.10)	0.49 (0.22, 1.10)
4-6 years	1.21 (0.87, 1.69)	1.24 (0.79, 1.94)	1.03 (0.46, 2.32)	1.03 (0.46, 2.33)
>6 years	2.03 (1.39, 2.96)	2.47 (1.45, 4.19)	1.16 (0.39, 3.45)	1.16 (0.39, 3.45)
# lifetime partners				
1	1.0	1.0	1.0	1.0
2	1.66 (1.29, 2.14)	1.21 (0.80, 1.82)	1.05 (0.43, 2.57)	1.05 (0.43, 2.57)
3	1.54 (1.04, 2.26)	1.25 (0.69, 2.28)	1.99 (0.67, 5.89)	1.99 (0.67, 5.89)
≥4	2.02 (1.54, 2.66)	1.98 (1.35, 2.91)	1.97 (0.87, 4.45)	1.97 (0.87, 4.45)
Primary partner had sex w/others:				
No	1.0	1.0	1.0	1.0
Yes	1.42 (1.05, 1.91)	1.35 (0.89, 2.04)	2.32 (0.98, 5.50)	2.32 (0.98, 5.50)
Don’t Know	1.29 (1.02, 1.64)	1.34 (0.94, 1.91)	1.48 (0.69, 3.15)	1.48 (0.69, 3.15)
Chlamydia infection ever:				
No	1.0	1.0	1.0	1.0
Yes	1.32 (1.04, 1.66)	1.74 (1.27, 2.38)	1.25 (0.54, 2.92)	1.25 (0.54, 2.92)
Bacterial Vaginosis at enrollment:				
No	1.0	1.0	1.0	1.0
Yes	2.11 (1.54, 2.91)	1.98 (1.28, 3.06)	2.28 (0.96, 5.39)	2.28 (0.96, 5.39)

*COC = Combined oral contraception.

**All variables mutually adjusted for in final model.

## Discussion

We identified a high prevalence of any HPV and any HR-HPV infection in a large population-based study of women recruited from seven family planning clinics across different geographic regions of Thailand. HPV 52 was identified as the most common HPV type. A higher prevalence of HPV was found to be associated with long-term use of combined oral contraceptives and prior and current diagnosis of sexually transmitted diseases, which appeared to remain significant after adjustment for sexual behavior. This study represents one of the largest and most comprehensive evaluations of HPV prevalence and correlates of infection in Thailand, a country where cervical cancer is one of most common cancers of women of reproductive age and a significant source of cancer-related mortality.

HPV 52 was the most common HPV type detected among women both with and without cervical abnormalities with a prevalence of 63% among women diagnosed with prevalent CIN2/3 as compared to only 27% for HPV 16. This elevated prevalence of HPV 52 decreased to only 45% after exclusion of women with HPV 16 co-infection. The relatively high prevalence of HPV 52 relative to HPV 16 among those with prevalent CIN 2/3 stands in contrast to a recent hospital-based study of 100 cervical tissue specimens collected from women with HSIL that reported 44.2% and 11.8% attributed to HPV 16 and HPV 52, respectively [12]. Part of this difference could be explained by the fact that our study used cervical swab to detect HPV as compared to biopsied cervical tissue. This may have led to detection of HPV 52 infections that are truly not associated with CIN 2/3. However, prior population-based studies conducted in Thailand that measured HPV in cervical and vaginal samples identified HPV 52 as the dominant type among women with a diagnosis of CIN 2/3 [5,13]. Other population-based studies assessing HPV prevalence in low- and middle- income countries show a high level of variability in the dominance of specific HPV types, particularly among cytologically normal women, as compared to high-income countries in Europe and the US where HPV16 is the most common type detected [14,15]. A meta-analysis utilizing HPV genotype data from 115,789 HPV positive women from different geographic regions with normal cytology, low and high grade pre-cancer, and invasive cancer observed a higher prevalence and potential greater contribution of HPV52 among women with normal cytology and women with low and high grade neoplasia as compared to other non-HPV16 types, particularly in East Asian populations [16]. However, the relative contribution of HPV52 to CIN3 and invasive cancer was less robust than other oncogenic HPV types. These results suggest that, in East Asian populations, HPV52 may be playing a role in early stage neoplastic transformation but its role in the progression to CIN3 and invasive cancer could be potentially less important than other oncogenic HPV types. This observation provides impetus for future work exploring the epidemiology and impact of other, non-HPV 16 oncogenic HPV types on cervical disease in middle and low income settings.

Long-term use of combined oral contraceptives is associated with an increased risk of cervical cancer diagnosis [17]. In Thailand, data from the IARC has estimated that combined oral contraceptive use is attributed to 23.1% of all cervical cancer cases [18]. Current and long-term combined oral contraceptive use has also been shown to increase risk of prevalent HPV infection among women <30 years of age [19-21]. However, longitudinal studies conducted to assess the association of COC use with HPV acquisition, persistence, and progression to pre-cancerous lesions have been inconsistent [22-29]. A detailed cross-sectional study conducted in this population confirmed a higher prevalence of any HPV and any HR-HPV infection among long-term COC users [21]. This finding agrees with prior longitudinal analyses which revealed an increased risk of HPV persistence as compared to an increased risk of HPV acquisition among current COC users [22]. Similar analyses conducted among DMPA users in this study population did not show any association with HPV prevalence, incidence or persistence in this population, lending to the specificity of the association. A variety of mechanisms have been proposed to help explain the potential role of sex steroid hormones on the natural history of HPV including (a) enhancement of cervical ectopy leading to enhanced acquisition of HPV; (b) modulation of host immune response by sex steroid hormones facilitating HPV persistence and development of cervical pre-cancer; (c) facilitating progression of an already established pre-cancerous lesion to invasive disease [30]. Additional studies in populations from other geographic regions are needed to clarify the epidemiologic association between COC use and HPV to strengthen the mechanistic hypothesis.

We observed higher prevalence of HPV infection among women diagnosed with either a current or prior history of bacterial vaginosis or Chlamydia Trachomatis. These associations remain significant even after adjustment for sexual behavior. Bacterial vaginosis is characterized as an alteration of the vaginal microflora that can result in inflammation and significant morbidity. Bacterial vaginosis has been suggested to increase the risk of HPV acquisition, presumably through disruption of non-specific physical immune barriers by alteration of vaginal pH [31]. A recent prospective study of over 9000 women from Costa Rica has shown that increases in vaginal pH were positively associated with HPV infection [32]. Chlamydia Trachomatis is one of the most common sexually transmitted infections worldwide, second only to HPV, and is associated with complications such as pelvic inflammatory disease, cervicitis, ectopic pregnancy and infertility. Chlamydia infection is associated with increasing the risk of HPV persistence, presumably through disruption of the mucosal immune response [33]. Although we cannot determine the temporal relationship of these genital tract infections on detection of HPV DNA in this study, our findings agree with other large cohort studies in where there was a similar detailed collection of sexual risk behavior and/or longitudinal analyses were conducted.

This study has several strengths. First, the use of highly sensitive and specific laboratory assays for HPV detection and genotyping allows for a higher degree of internal validity and better assessment of the outcome measures. The use of histological and cytological methods for diagnosis of CIN helps minimize potential over-reporting of disease endpoints. Second, given the study cohort was derived from a study assessing the effects of hormonal contraceptive use on HIV acquisition, detailed information regarding reproductive factors such as contraceptive usage history as well as sexual behavior and other risk factors were collected which allowed for a thorough investigation of these exposures on the risk of HPV prevalence.

Limitations of the study include the cross-sectional design which limits the ability to ascribe a temporal relationship between the factors examined and HPV outcomes. Prospective studies are therefore needed to address the association of these factors on endpoints of HPV such as acquisition and persistence. Additionally, it is important to note that sexual behavior information, in particular male partner behavior, was based on participant self-report and therefore may underestimate the prevalence of certain risky sexual behaviors that may increase the association with HPV infection. Finally, the generalizability of the study’s findings may be limited. The reported prevalence of any HPV and any HR-HPV in this study is about 4-times higher than previously reported population-based prevalence survey’s in Thailand using similar HPV detection and genotyping assay’s with similar levels of sensitivity [5,13]. The current study sampled women between the ages of 20–37 years from varied geographic settings in Thailand who were family-planning clinic attendees. These women may, therefore be at elevated risk of HPV exposure and infection relative to the general population. However, the similarity of HPV type distribution among both diseased and non-diseased women, particularly with respect to HPV 52, in this population as compared to previous studies lends strength to the relevance and generalizability of these findings in the broader context of the epidemiology of HPV in Thailand.

The current study demonstrates a uniquely high prevalence of HR-HPV types such as HPV 52 among women with cervical pre-cancer in Thailand. These data provide descriptive information for development and implementation of prophylactic interventions, including vaccine formulations that target a broader spectrum of oncogenic HPV types.

## Conclusions

The prevalence of any HPV and HR-HPV in this sample of high risk women attending family planning clinics throughout Thailand is 24.6% and 14.0% with HPV type 52 being the most common HPV type among women with both normal and abnormal cervical cytology and women with biopsy confirmed CIN 2/3. The prevalence estimates of any HPV type and HPV 52 in this population could be used update regional and country-specific estimates on the burden of HPV among healthy women and women with low and high grade cervical disease. Long-term use of hormonal contraception, laboratory confirmed Chlamydia infection, and bacterial vaginosis was associated with an elevated prevalence of any HPV and any HR-HPV. The identification of these specific and modifiable risk factors could help guide secondary prevention measures for cervical cancer such as targeted screening programs. Overall these study results call for additional, longitudinal studies on the natural history of HPV in Thailand where cervical cancer is one of the most important contributors of cancer-related morbidity and mortality among women of reproductive age.

## Additional file

Additional file 1: Table S1. Multivariate association of factors with prevalent infection of any HPV and any HR-HPV by Pap Smear Status.

## Abbreviations

HPV: Human Papillomavirus
HR-HPV: High Risk Human Papillomavirus
COC: Combined oral contraception
DMPA/DepoPrevera: Depomedroxyprogesterone Acetate
GEE: generalized estimating equation
