# Supplementary material for: Prevalence and correlates of HPV among women attending family-planning clinics in Thailand
Source: BMC Infect Dis. 2015 Mar 27;15:159. doi: 10.1186/s12879-015-0886-z (PMC4387719; doi:10.1186/s12879-015-0886-z)
Supplement: Additional file 1: Table S1. — Multivariate association of factors with prevalent infection of any HPV and any HR-HPV by Pap Smear Status. [file 12879_2015_886_MOESM1_ESM.docx]

Additional file 1: Table S1: Multivariate association of factors with prevalent infection of any HPV and any HR-HPV by Pap Smear Status

|  | Normal Cytology (N= 1050) | | Abnormal Cytology (N=121) | |
| --- | --- | --- | --- | --- |
| Variable | Adjusted PR (95% CI)** | | Adjusted PR (95% CI)** | |
|  | Any HPV | Any HR-HPV | Any HPV | Any HR-HPV |
| Age at enrollment: |  |  |  |  |
| <26 | 1.0 | 1.0 | 1.0 | 1.0 |
| 26-30 | 0.92 (0.66, 1.29) | 0.84 (0.53, 1.32) | 0.95 (0.65, 1.39) | 0.87 (0.52, 1.45) |
| 31-33 | 1.04 (0.72, 1.50) | 0.78 (0.47, 1.30) | 0.89 (0.59, 1.33) | 0.89 (0.52, 1.53) |
| 34-38 | 0.99 (0.69, 1.43) | 0.83 (0.51, 1.37) | 0.77 (0.48, 1.25) | 0.89 (0.51, 1.59) |
|  |  |  |  |  |
| Cumulative use of COCs*: |  |  |  |  |
| Never | 1.0 | 1.0 | 1.0 | 1.0 |
| <4 years | 1.16 (0.79, 1.69) | 1.16 (0.69, 1.96) | 1.07 (0.69, 1.63) | 0.88 (0.55, 1.40) |
| 4-6 years | 1.10 (0.72, 1.68) | 1.19 (0.66, 2.17) | 1.15 (0.73, 1.82) | 0.91 (0.53, 1.54) |
| >6 years | 1.99 (1.27, 3.12) | 2.98 (1.64, 5.42) | 1.66 (0.97, 2.84) | 1.14 (0.56, 2.33) |
|  |  |  |  |  |
| # lifetime partners |  |  |  |  |
| 1 | 1.0 | 1.0 | 1.0 | 1.0 |
| 2 | 1.87 (1.40, 2.49) | 1.76 (1.17, 2.64) | 1.08 (0.67, 1.72) | 0.93 (0.51, 1.72) |
| 3 | 1.45 (0.86, 2.45) | 2.02 (1.10, 3.71) | 1.94 (1.27, 2.96) | 1.58 (0.79, 3.13) |
| >4 | 2.50 (1.84, 3.41) | 2.52 (1.63, 3.87) | 1.44 (0.98, 2.09) | 1.48 (0.91, 2.41) |
|  |  |  |  |  |
| Primary partner had sex w/others: |  |  |  |  |
| No | 1.0 | 1.0 | 1.0 | 1.0 |
| Yes | 1.51 (1.04, 2.19) | 1.85 (1.16, 2.95) | 1.33 (0.89, 1.96) | 1.55 (0.97, 2.48) |
| Don’t Know | 1.18 (0.87, 1.59) | 1.30 (0.86, 1.97) | 2.23 (1.63, 3.06) | 2.37 (1.56, 3.61) |
|  |  |  |  |  |
| Chlamydia infection ever: |  |  |  |  |
| No | 1.0 | 1.0 | 1.0 | 1.0 |
| Yes | 1.41 (1.07, 1.88) | 1.67 (1.16, 2.41) | 1.12 (0.68, 2.01) | 1.53 (0.99, 2.36) |
|  |  |  |  |  |
| Bacterial Vaginosis |  |  |  |  |
| No | 1.0 | 1.0 | 1.0 | 1.0 |
| Yes | 2.41 (1.67, 3.49) | 2.37 (1.43, 3.93) | 1.17 (0.68, 2.01) | 1.07 (0.63, 1.81) |
| *COC = Combined oral contraception  **All variables mutually adjusted for in final model | | |  |  |
